# Use of Customizable Nucleases for Gene Editing and Other Novel Applications

**DOI:** 10.3390/genes11090976

**Published:** 2020-08-22

**Authors:** Pradeep Reddy, Felipe Vilella, Juan Carlos Izpisua Belmonte, Carlos Simón

**Affiliations:** 1 Gene Expression Laboratory, Salk Institute for Biological Studies, La Jolla, CA 92037, USA; belmonte@salk.edu; 2 Igenomix Foundation, Instituto de Investigación Sanitaria Hospital Clínico (INCLIVA), 46010 Valencia, Spain; felipe.vilella@igenomix.com (F.V.); carlos.simon@igenomix.com (C.S.); 3 Department of Obstetrics and Gynecology, BIDMC, Harvard University, Boston, MA 02215, USA; 4 Department of Pediatrics, Obstetrics and Gynecology, School of Medicine, University of Valencia, 46010 Valencia, Spain; 5 Department of Obstetrics and Gynecology, Baylor College of Medicine, Houston, TX 77030, USA

**Keywords:** genome editing, genetic diseases, embryos

## Abstract

The development of novel genome editing tools has unlocked new opportunities that were not previously possible in basic and biomedical research. During the last two decades, several new genome editing methods have been developed that can be customized to modify specific regions of the genome. However, in the past couple of years, many newer and more exciting genome editing techniques have been developed that are more efficient, precise, and easier to use. These genome editing tools have helped to improve our understanding of genetic disorders by modeling them in cells and animal models, in addition to correcting the disease-causing mutations. Among the genome editing tools, the clustered regularly interspaced short palindromic repeats (CRISPR)/CRISPR-associated (Cas) system has proven to be the most popular one due to its versatility and has been successfully used in a wide variety of laboratory animal models and plants. In this review, we summarize the customizable nucleases currently used for genome editing and their uses beyond the modification of genome. We also discuss the potential future applications of gene editing tools for both basic research and clinical purposes.

## 1. Introduction

The ability to precisely manipulate the genome revolutionized not only molecular biology, but also opened several areas of biotechnology that are useful for research, agriculture, and medicine. Initial gene targeting primarily depended on homologous recombination to insert exogenous DNA sequences in the human cells [1]. The establishment of mouse embryonic stem (ES) cells led to the application of homologous recombination in pluripotent cells to modify the genome and generation of genetically engineered mice [2,3,4]. Initially, the efficiency of gene targeting using homologous recombination was extremely low, which was solved to an extent by enriching the edited cells using the antibiotic selection cassette. Interestingly, Russell and Hirata observed that recombinant adeno-associated virus (rAAV) vectors with homology arms proved efficient in modifying the chromosomal target sequences [5]. The mechanism behind this observation is not entirely clear. However, the single-stranded DNA of the AAV genome and higher transduction rates might be the reason for higher homology recombination. 

Double-strand breaks (DSBs) on DNA are repaired using three different mechanisms, namely non-homology end joining (NHEJ), microhomology-mediated end joining (MMEJ), and homologous recombination (HR). Due to the nature of the repairs, NHEJ and HR are referred to as error-prone and error-free, respectively. NHEJ is active during all the cell cycle phases, whereas HR occurs only during the S-G2 phase of the cell cycle. For this reason, NHEJ is active in all the cell types, both dividing and non-dividing. During the repair of DSBs, the donor template with homology arms can get inserted at the site of repair using homology-directed repair (HDR) and dramatically increases the gene targeting efficiency [6]. Based on this method, rare cutting endonucleases such as the 18-bp cutter I-SceI have been used to introduce DSBs to increase the gene targeting efficiency [7]. Despite the presence of several natural meganucleases with unique recognition sites, the chances of finding a cutting site at the desired location are low. Nevertheless, modifications to meganucleases allowed some of the challenges to be overcome [8]. 

The discovery of customizable nucleases that can be programmed to induce DSBs at desired loci on the genome dramatically increased the efficiency of homologous recombination, leading to another revolution in gene editing with much broader implications in several different fields. This review aims to provide an overview of the recent developments in and applications of engineered nucleases that have helped lay the groundwork for their use not only for genome editing in various animal models, but also to correct the genetic mutations in human cells for clinical use. We also discuss the additional applications of genome editing tools in addition to modifying the genome, such as modulating the expression of genes and live-cell imaging. Finally, we review the use of genome editing tools in human embryos. 

## 2. Customizable Nuclease

### 2.1. Zinc-Finger Nucleases (ZFNs)

ZFNs are a class of DNA-targeting components with two monomer subunits containing DNA-binding and cleavage domains. Each monomer is composed of three zinc fingers, which recognize nine base pairs and a *Fokl* endonuclease domain. The DNA-binding zinc fingers can be engineered to bind to specific regions on the genome. *Fokl* is enzymatically active as a dimer; therefore, two ZFN subunits are designed for each target sequence to facilitate the dimerization and cleavage [9]. The dimerization requirement of *Fokl* and binding of fingers to the target sequence have increased ZFNs attractiveness as a tool for introducing DSB at desired locations on the genome, which is mostly repaired by NHEJ pathway, resulting in the disruption of the amino acid sequence and function of the gene [10,11]. ZFNs have been widely used to create mutations in a wide variety of organisms, including, drosophila, zebrafish, mouse, rats, sea urchins and frogs [11]. ZFNs have also been employed for therapeutic purposes, such as disrupting the *CCR5* gene to interrupt the expression of co-receptor and prevent HIV infection [12]. Moreover, homologous recombination with donor template DNA at the target site has also been achieved in mice and rat embryos [13,14]. However, gene targeting did not succeed in all the animals, such as zebrafish, despite a high rate of DNA cleavage and mutagenesis, potentially due to differences in DNA repair mechanisms [11]. ZFNs are smaller in size and are presumed to possess low immunogenic properties due to their similarity to mammalian transcription factors. Nevertheless, the complexity and cost of designing the domains are disadvantages of ZFNs. 

### 2.2. Transcription Activator-Like Effector Nucleases (TALENs)

Similar to ZFNs, TALENs have a customizable DNA-binding domain and *FokI* nuclease domain. The TALE-DNA-binding domain is composed of conserved repeats domains derived from transcription activator-like effectors (TALEs) secreted by *Xanthomonas* bacteria to alter the transcription in host cells [15]. Each TALE repeat binds to a specific single base of DNA, and the number of repeats corresponds to the length of the target site. Two TALE repeats with target site binding domains fused with the catalytic domain of *FokI* endonuclease are used to create a DSB. TALENs exhibit higher specificity and efficiency than ZFNs. For this reason, TALENs have been widely used for genetic manipulation in different organisms [16]. Additionally, two pairs of TALENs have been used on the same chromosome to generate large deletions [17]. Moreover, TALENs have also been employed to introduce the donor sequences into the genome [18]. The single base pair recognition of TALE allows greater flexibility in designing the TALENs; however, the repeat arrays of TALE presents a technical challenge in cloning the identical sequences and also delivering in viral vectors. Additionally, the large size and immunogenicity of TALENs limit their clinical applications. 

### 2.3. CRISPR-Cas System

The clustered regularly interspaced short palindromic repeats (CRISPR)/CRISPR-associated 9 (Cas9) nuclease is a recently identified system in prokaryotes with adaptive immunity against viruses and plasmids. Cas9 complexes with CRISPR RNA (crRNA) and trans-activating crRNA (tracrRNA) to form an endonuclease that can recognize and cleave foreign genetic sequences. The DNA binding occurs using a 20-base pair DNA sequence in the crRNA that complements the targeting region next to protospacer adjacent motif (PAM) that triggers Cas9 to create DSB. During the repair of DSB, primarily by NHEJ, small indels are created at the target site, which results in deletions, insertions or frameshift mutations leading to loss of function of the gene. The sequence of crRNA can be replaced with any synthetic target sequence without modifying the other components. The crRNA and tracrRNA were fused to form a single chimeric RNA (sgRNA) and complexed with Cas9 to induce site-specific DNA cleavage [19]. The applicability of CRISPR-Cas9 for genomic modifications was demonstrated in human cells [20,21,22,23]. The replacement of only the targeting sequence in the RNA component and higher targeting efficiency made the CRISPR-Cas9 an attractive genome editing tool. Moreover, the simplicity in design and cloning facilitated the adoption of the system to various labs around the globe. Notably, the size of the Cas9 nuclease also advantageously allowed the delivery of the system in vivo using adeno-associated viruses (AAVs) [24,25,26,27,28,29]. Recently, additional Cas9 orthologues that recognize different PAM sequences have been discovered [30]. The CRISPR-Cas9 has been widely used for genetic modification in several different organisms, including plants [31,32]. Importantly, the clinical potential of CRISPR-Cas9 to correct the genetic mutations that result in the manifestation of the diseases is demonstrated in several different animal models [31]. However, the presence of antibodies against certain types of Cas enzymes and a distinct cellular and molecular changes in the host raise concerns [33,34,35,36]. Thus, large-scale screening for antibodies in different populations and further studies in larger animals are required to reach a conclusion.

In an adult mammal, the majority of the cells in the body are post-mitotic, with a few exceptions like cells in the liver. The classical HDR mechanism is generally inefficient in non-dividing cells, which limits the possibility of performing a knock-in in these cells. We developed an NHEJ-based homology independent strategy using CRISPR-Cas9, named homology-independent target integration (HITI), for the integration of transgene in non-dividing cells [29,37]. Unlike the HDR-based CRISPR-Cas9 approach, the transgene that is integrated using the HITI method lacks homology regions but harbors Cas9 cleavage sites similar to the targeting sequence on the genome. The cleavage by Cas9 creates blunt ends on the transgene and at the targeting site, while the NHEJ pathway allows the integration of transgenes during the repair of genomic DSB. As the NHEJ pathway is active during all cell cycle phases, HITI opened the doors for targeted gene knock-in in non-dividing cells, including neurons and muscle [29]. However, HITI cannot repair existing mutations; therefore, it is limited in its ability to correct point or frameshift mutations. Recently, we developed another versatile knock-in method called intracellular linearized single homology arm donor mediated intron targeting integration (SATI) for gene knock-in in intronic regions using donor vector containing a single homology arm with a Cas9 cleavage site [38]. SATI has a bipotential capacity to use HDR and NHEJ DSB repair machinery, facilitating in targeting a broad range of mutations in different cell types. SATI was successfully used for gene knock-in in vivo to correct a dominant point mutation that causes premature aging in mice [38]. Similarly, several other gene-editing methods utilizing CRISPR-Cas9 and MMEJ were developed during the last couple of years for both in vitro and in vivo application [37].

In addition to the use of CRISPR-Cas9 for gene deletion and insertion, Cas9 nickase and catalytically deficient Cas9 (dCas9) are fused with deaminases for the conversion of single nucleotides. The cytosine base editor (CBE) is the first base editor developed that can convert C to T [39]. The commonly used third-generation CBE consists of a cytidine deaminase (APOBEC1), Cas nickase, and a uracil glycosylase inhibitor (UGI). In mammalian cells, an average of 37% permanent conversion at the target site is reported [39]. Nishida et al. also reported another cytosine base editing system using cytidine deaminase 1 (CDA1) [40]. Later, adenine base editor (ABE) was developed by replacing the cytidine deaminase with adenine deaminase (TadA) that can convert A to G [41]. The ABE comprises heterodimeric proteins (wild-type non-catalytic TadA monomer and evolved TadA* monomer) and Cas9 nickase in a single polypeptide chain. Base editors are successfully used in plants, zebrafish, mice and human embryos [30]. Additionally, RNA base editors (RBE) are created by fusing nucleobase deaminase with Cas13 protein to convert A to inosine (I) or C to uracil (U) in the targeted RNA [42].

Interestingly, three independent groups have recently reported the development of dual adenine and cytosine base editors by combining both the deaminases with Cas9 nickase [43,44,45]. Using the dual deaminase base editor, both A to G and C to T substitutions were achieved at the target site. The dual deaminase base editors will offer new possibilities that were not possible with single-deaminase base editors such as targeting multi-nucleotide variants and CA/TG-rich transcription factor binding sites [43]. Additionally, Zhao et al. developed glycosylase base editors that can cause C to A transversions in bacteria and C to G transversions in mammalian cells [46]. The glycosylase base editors contain Cas9 nickase, a cytidine deaminase and uracil-DNA glycosylase. In mammalian cells, editing efficiency of 5–53% was observed. 

Base editors do not generate DSBs and the chances of undesired changes such as indels are low. However, the base editors sometimes show off-target specificity [47]. Moreover, the presence of multiple C and A nucleotides close to the target base called “bystander base” can result in multiple base conversions, which can affect the targeting efficiency of base editors. Notably, Arbab et al. reported the creation of a machine learning model called BE-Hive using experimental data from more than 38,000 target sites in human and mouse cells [48]. This tool facilitates in predicting the base editing efficiency at the target site, especially in the presence of bystander nucleotides. 

Anzalone et al. reported a prime editing approach similar to base editors, where Cas9 nickase was fused to an engineered reverse transcriptase, which is guided by a two-part guide RNAs termed “search” and “replace”. The “search” guide localizes the Cas9 to target loci, where it cuts the DNA and the “replace” guide sequence is used by reverse transcriptase to make complementary DNA to integrate at the cut site [49]. Since most of the genetic diseases occur due to point mutations, the therapeutic potential of base and prime editors for the treatment of human disorders is currently being explored [50,51,52]. 

Interestingly, the CRISPR-Cas complex was also found to be encoded in a class of bacterial Tn7-like transposons [53]. Due to the site-specific integration of Tn7 transposon downstream of the conserved genomic sequence in *Escherichia coli*, researchers have hypothesized that transposon encoded CRISPR-Cas promoted this process [53]. In a recent study, Klompe et al. demonstrated how transposons have used the RNA-guided DNA targeting mechanism for site-specific integration without the need of DSBs and homologous recombination [54]. The unique features of this system might help to overcome the issues that arise from potential DSBs at off-target sites and the requirement of a long-homology arm, which limits the size of the target sequence. However, the system has not been tested in human cells and the efficiency of integration of template DNA at the target site remains unknown [55].

In addition to the targeting of DNA, Cas9 is also directed to target the single-stranded RNA (ssRNA) matching the guide RNA sequence when PAM is presented in trans as a separate DNA oligonucleotide (PAMmers). The PAMmers were able to stimulate the Cas9 endonuclease activity on ssRNA, similar to DNA cleavage [56]. This approach was used to eliminate the toxic microsatellite repeat expansion RNA in myotonic dystrophy type 1 patient cells [57]. Later, a naturally existing CRISPR-Cas13 system with RNA-targeting endonuclease activity was discovered in bacteria. Cas13 has four family members (Cas13a-d) and each demonstrates specific base preferences; for example, Cas13a requires a protospacer flanking sequence consisting of a single A, U or C base pair [58]. Moreover, Cas13 subtypes differ in CRISPR RNA structure, direct repeat sequence and size. Although all exhibit promiscuous ribonuclease activity upon target recognition, Cas13d RNA cleavage is observed only in bacteria and not in the mammalian cells [59]. This unique characteristic of Cas13d allowed targeting RNAs in mammalian cells [59,60].

## 3. Specificity and Off-Target Effects of Customizable Nucleases

The specificity of nucleases used for genome editing is one of the essential criteria for success in both basic and translational research. The customizable nucleases are engineered to target the region of interest on the genome using sequence information; however, the shorter-targeting sequence length used for the identification and few mismatches might allow the binding of nucleases at off-target sites. The concern of off-target applies to all the customizable nucleases in general. For therapeutic use of nucleases, they need to demonstrate higher specificity, activity, and gene modification ability. They should also be easier to deliver. Along this line, re-engineering strategies have been implemented for Zinc finger proteins and TAL effectors to improve the targeting precision [61,62,63]. Since Zinc finger nucleases and TALENs have been actively used for a long time, they demonstrate a long track record in safety, including their use in patients for therapeutic purposes. Conversely, CRISPR is relatively new; due to the simplicity in its design and ease of use, it is widely used, but we are still understanding the specificity and repair outcomes [63]. In the initial studies using CRISPR-Cas9, depending on the number of mismatches in the guide RNA, the rate of off-target effects were observed [20,64,65]. Later, a Cas9 nickase mutant paired with two guide RNAs was used to create DSBs, which led to reduction of off-target effects by 50 to 1000-fold [66]. Similarly, base and prime editing approaches can be a good alternative way to correct the disease-causing mutations without introducing DSBs and thereby avoid unintended changes. Nevertheless, the development of advanced bioinformatics tools and high-throughput sequencing methods will help in screening and identification of guide RNAs that are specific and do not have any off-targets [67]. In addition, the use of cell- or tissue-specific promoters to restrict the expression of CRISPR in combination with the local delivery, short-term expression, and use of selective AAV serotype can also help to minimize the negative effects. 

## 4. Applications of Customizable Nucleases

### 4.1. Modeling Genetic Diseases in Cells

The traditional approach of genetic modification in the cells is less efficient, especially in performing targeted gene knockout or knock-in of fluorescent reporters. The customizable nucleases allowed the generation of a variety of genetic modifications in mammalian cells to model diseases, including cancer, metabolic and neurodegenerative diseases [68]. Stem cells are routinely used in the laboratories as they can be differentiated to any cell type using a defined medium, but genome editing by homologous recombination is less efficient and possesses limited use in the modeling diseases. The generation of pluripotent stem cells from patient somatic cells by reprogramming opened the possibilities of generating personalized induced pluripotent stem cells (iPSCs) [69]. iPSCs are routinely used to generate disease causing mutations to mimic the disease phenotypes in cell culture, which aid in understanding the cellular and molecular changes that occur during the disease progression [70]. Also, the engineered iPSCs can be differentiated to other cell types and used them for large-scale drug screenings and CRISPR knockdown screenings to identify new gene networks that play a role in disease progression or prevention. Moreover, pluripotent stem cells are widely used to generate different types of organoids [71,72]. Interestingly, gene-editing tools are also used in the in vitro organoid system to model human diseases to understand the etiology of the disease as well as for drug screening [73]. Notably, the genetically engineered cells have been used to generate several different types of organoids, including the brain, intestine, liver, kidney and lung [70].

### 4.2. Correction of Disease-Causing Mutations

The correction of genetic mutations to treat human diseases has proven an exciting potential application for the genome editing tools. In this line, ZFNs, TALENs, and the CRISPR-Cas system have been used to correct mutations in cells in vitro and transplanting them back to the patients. ZFNs have been successfully employed in mammalian cells to deliver the vectors expressing the ZFN-coding sequences by transfection of DNA or infection with viruses [11]. The higher specificity and mutagenesis rate of ZFN provides an advantage for therapeutic applications. ZFNs have been used to knock out the *CCR5* gene in T-cell precursor cells isolated from HIV patients to avoid the infection of HIV-1 [12]. Currently, clinical trials are ongoing that use this approach with modifications in the delivery of ZFNs to T-cells and improvements in the engraftment of infused cells [74]., Moreover, the genetic mutation that causes β-thalassemia and sickle cell disease in hematopoietic stem cells has been corrected using ZFNs [75,76]. Similarly, TALENs are also used in chimeric antigen receptor (CAR) T-cell therapy, where the T-cells are genetically modified to produce artificial receptors that recognize a specific protein on the tumor cells. Many products based on TALEN-edited cells have begun clinical trials, especially those focused on immuno-oncology [74]. 

Similar to ZFN and TALEN, T-cells have been genetically edited using CRISPR to knockdown immune checkpoint inhibitor programmed cell death-1 (PD-1), which is upregulated during activation of T-cells to reduce the autoimmune reaction and aid cancer cells in evading the immune system [77]. Moreover, CRISPR is also used to induce exon skipping of defective exons in Duchenne muscular dystrophy (DMD), to inactivate faulty genes that lead to the manifestation of disease including amyotrophic lateral sclerosis and Huntington disease, correct a genetic mutation that causes premature aging and eliminate the entire chromosome in aneuploid stem cells [32,78,79]. Importantly, gene-editing tools have already been used to correct the mutation in *β-globin* gene in patient hematopoietic stem cells [31].

ZFNs and CRISPR-Cas9 have been used to edit the HIV-1 genome and block its expression in T-cells, microglia and promonocytes [80,81,82]. CRISPR-Cas9 has been used to directly target and disrupt the reverse-transcribed products of lentivirus RNA generated during their life cycle [83]. Although the use of nucleases to edit the HIV-1 genome from human cells may not be considered a correction of disease-causing mutations, the HIV-1 genome in the cells produces new viral particles that lead to the development of acquired immune deficiency syndrome (AIDS). 

The recent development of the NHEJ based genome-editing approach in non-dividing cells now allows for correcting mutations that cover a wide range of diseases, including Parkinson’s disease and amyotrophic lateral sclerosis. HITI was employed to correct a mutation that causes inherited degenerative eye disease, retinitis pigmentosa, in rats [29]. Likewise, SATI was used to knock-in a normal copy of *Lmna* and prevent the expression of mutated copy, which led to the extension of the lifespan of progeria mice [38]. Furthermore, other NHEJ and CRISPR-Cas9 based approaches were used to knock-in a normal copy of the gene to rescue diseases such as tyrosinemia type I in mice [37]. Notably, several clinical trials are ongoing using CRISPR for the treatment of genetic diseases, including a blood disorder, eye disease and muscular dystrophy [31]. Likewise, CRISPR single-base editors are now used for the correction of single-base mutations or to disable the expression of the mutant gene [52]. Although CRISPR-based gene editing was identified recently, it has been tested in almost all cell types.

### 4.3. Generation of Animal Models

The traditional approach for creating gene-edited animals requires a time-consuming method that involves the use of modified ES cells. The generation of modified ES cells is a time and labor-intensive process. Moreover, the low success rate of obtaining the founder mice with a higher contribution of injected ES cells to the germline also hindered the process. Furthermore, the lack of ES cells for certain animals, including rats, also limited the generation of genetically modified animals of different species. However, the development of customizable nucleases has helped to overcome the need for ES cells by performing the gene modifications directly in the zygotes. ZFNs were the first nucleases widely used for targeted mutagenesis and gene replacement, beginning with fruit fly and nematodes and progressing to other organisms such as silkworm and zebrafish, by injecting the ZFN pair at the early stages of zygote or embryo development. Notably, ZFNs opened the door to the possibility of creating genetically modified rats, which was not possible with other approaches [84]. In mice and rats, ZFNs have been able to induce both mono and biallelic gene disruption and homologous recombination with donor DNA at the target site. Furthermore, the germ cells carried the modifications. Similar to ZFNs, TALENs have been used to disrupt the expression of target genes in different animal models, including mice, rats, frog, zebrafish, pig and fruit fly [16]. In most of these studies, a single TALEN pair was used to create DSB to interrupt the function of the targeted gene. Moreover, to generate large deletions or chromosomal inversions, two pairs of TALENs were used to target the same chromosome [85,86]. The higher genome editing efficiency of TALEN is also used for generating animal models that mimic human diseases such as hypercholesterolemia.

Currently, the CRISPR-Cas system is the most favored customized nuclease and is widely used for performing in vivo genetic modification. Importantly, the CRISPR-Cas system allows the possibility of the simultaneous manipulation of several different genes, thereby dramatically reducing the amount of time required for the generation of double- or triple- knockout animals [87]. Moreover, in addition to mice, CRISPR has been successfully used in rats and other large animals, including dogs, pigs and monkeys [88]. In addition to performing gene knockout in vivo, the CRISPR-Cas system is also used to perform knock-in of reporters not only in dividing cells but also in non-dividing cells such as neurons [29,37]. Likewise, CRISPR-Cas9 along with NHEJ and MHMEJ-based methods have been used for transgene knock-in in mouse and monkey embryos [89]. Wang et al. reported the generation of non-human model of Hutchinson-Gilford progeria syndrome (HGPS) in monkey embryos using base editor to introduce C to T conversion in *LMNA* gene [90]. However, the microinjection of nucleases into zygotes requires special equipment and skill, which are not always available in all labs. To solve this problem, alternative methods are being developed, such as the injection of nucleases into the oviduct of pregnant females, followed by in vivo electroporation to deliver the components into zygotes [91].

### 4.4. Targeting Mitochondrial Genome

Among the different nucleases, so far, ZFNs and TALENs are the only ones so far that have been successfully used to target the mitochondrial genome. Unlike the nuclear genome, mitochondrial DNA (mtDNA) is mostly maternally inherited. The number of mitochondria and mtDNA can vary between different cell types and tissues. Moreover, multiple copies of mtDNA exist, ranging from a few hundred to thousands in each cell based on their energy demand. The mutations in the mtDNA lead to the degeneration of tissues and organs with high energy demand, including muscle and neurons, resulting in the manifestation of mitochondrial disease phenotypes [92]. Due to a lack of an efficient DNA repair mechanism in the mitochondria and the presence of multiple copies of both mutated and non-mutated mtDNA, the strategy of selective elimination of mutated mtDNA and re-population of normal mtDNA has been undertaken [93]. ZFNs and TALENs are localized into the mitochondria using a mitochondria localization signal and they selectively degrade the mtDNA with the disease-causing mutation in the cells obtained from mitochondrial disease patients [94,95,96]. Moreover, in a recent study, both ZFN and TALENs were delivered in vivo to reduce the mtDNA with a disease-causing mutation in muscle and heart and rescue the disease phenotypes in a mitochondrial disease mouse model [97,98]. Additionally, TALENs were also used in the mice embryos to target specific mitochondrial haplotypes and prevent their transmission to the next generation [99]. 

However, the CRISPR-Cas system has not been reported to be successfully used for targeting mtDNA. The major hurdle for the application of CRISPR-Cas system to mtDNA editing has been the successful import of gRNAs into the mitochondrial matrix, partially due to the inefficient RNA import mechanism present in the mitochondria of mammalian cells [100]. Although a few publications report the use of CRISPR-Cas9 to target mtDNA, the results presented in the studies lack proper experimental evidence and have not yet been independently validated by other groups [101,102]. Interestingly, a new study reports a CRISPR-free mitochondrial base editing approach, which is accomplished using an interbacterial cytidine deaminase toxin fused to TALE domains [103]. The cytidine deaminase was split into two halves to avoid the toxicity and is inactive until brought together at the target site. Using this new approach, the CG to TA base conversions in mtDNA are possible without the need of DSBs and five different genes on mtDNA were edited without any off-target effects [103]. Though this approach is promising to introduce base changes in the mtDNA, a strong preference for 5′-TC-3′ is reported, which dramatically limits the number of target regions. In fact, only one mitochondrial mutation 8356T>C found in humans can fulfill this requirement. Nevertheless, the modification of current cytidine deaminase may allow us to overcome this requirement. Currently, we lack animal models for mitochondrial disease due to the inability to edit the mtDNA. However, a CRISPR-free mitochondrial base editing approach can be used to generate animal models by introducing pathogenic mutations in the mtDNA.

## 5. Applications Beyond Genome Editing

### 5.1. Epigenetic Modifications

Although initial applications of nucleases mostly focused on gene editing, they were quickly redirected for other purposes, such as gene regulation. Zinc-fingers and TALEs were fused to transcriptional activators, among the different activators, VP64 and p65 proved the strongest when targeted upstream of the transcription start site and in promoter regions [104]. Similarly, the catalytic inactive dCas9 was fused with different transcriptional activators, repressors, modifiers or fluorophores to modulate the expression of the target gene by modifying the epigenetic status at the promoter regions [30]. To repress the gene expression, dCas9 was fused with the transcription repressor domain Krüppel-associated box (KRAB), which induces heterochromatin formation and changes in chromatin structure. dCas9-KRAB has been shown to silence genes, non-coding RNA and proximal and distal enhancer elements [30]. For gene activation, multiple transcription activation domains were fused to dCas9 [105] or recruited using the dimerized MS2 bacteriophage coat proteins that bind to the minimal hairpin aptamer on the gRNA tetraloop and stem-loop 2 [106]. Using this approach, multiple genes were simultaneously activated at the same time for cellular reprogramming of somatic cells to pluripotent state or direct differentiation of fibroblasts to neurons [30]. Furthermore, other epigenetic modifications, such as the acetylation and methylation of DNA have also been performed by fusing histone acetyltransferase p300 or catalytic domain of methylcytosine dioxygenase TET1 to dCas9 [104]. Recently, a study achieved dose-dependent activation of genes was achieved using CRISPR and chemical epigenetic modifiers that recruit endogenous chromatin machinery [107]. 

We have shown, for the first time in vivo, that Cas9 and transcription activation complexes can be recruited to the target loci using modified guide RNAs to activate the expression of endogenous genes [108]. Using this approach, we treated acute kidney disease, diabetes and muscular dystrophy in mouse models [108]. Later, a transgenic mouse model expressing a modified dCas9 system was used in vivo to activate multiple neurogenic endogenous genes to directly convert astrocytes into functional neurons [109]. Recently, Matharu et al. delivered dCas9-VP64 using AAVs to rescue the obesity phenotypes in a haploinsufficient mouse model [110]. Moreover, CRISPR-Cas12a was fused to a transcriptional activation domain to enable multiplexed knockout and transcriptional activation in vivo [111]. Similarly, the CRISPR system was also used to repress the expression of genes in vivo. In the last few years, several studies have successfully delivered dCas9 fused with different transcriptional repressors to reduce the expression of genes that play a role in reducing brain function [112], lower circulating low-density lipoprotein (LDL) concentration [113], and to correct retinitis pigmentosa phenotypes [114]. All these studies demonstrate the therapeutic potential of CRISPR based epigenetic modifications in vivo in ameliorating disease phenotypes [115]. 

### 5.2. Live Cell Genome Imaging and Large-Scale Genetic Screenings

Among the different repurposes, nucleases were also used for labeling specific regions of chromatin using fluorescent proteins. Before the identification of CRISPR-Cas system, ZFN and TALE proteins were used to recruit the fluorescent protein to the regions such as telomeres and centromeres for live imaging in cells [116,117]. Higher targeting efficiencies of Cas9 and dCas9 have replaced the other nucleases. Moreover, in another study, the improvement to the gRNA scaffold containing multiple MS2 binding modules facilitated the increase of the fluorescent signal with as few as four gRNAs. This improvement facilitated better labeling of low-repetitive and non-repetitive regions and tracking of the transcriptionally active and inactive regions [118]. Among the different nucleases, CRISPR-Cas system is the popular choice for performing high-throughput screens. Additionally, the CRISPR-Cas system is also employed in large-scale functional screening using hundreds of gRNAs in the cells and is efficient compared to the traditional short hairpin RNA (shRNA) system [119]. This approach allows large-scale genetic screening in an unbiased way to understand the role of a gene on a specific phenotype [106,120,121]. CRISPR-Cas system is also used to perform large-scale knockout screens to identify causative drivers of cancer in a native tissue environment [122,123]. Notably, this type of screen can be performed in a regular laboratory with access to cell culture, and no automated robotics are required. However, it involves a significant amount of sequencing, data analysis, and validation of data [124].

### 5.3. Use of CRISPR to Detect Nucleic Acids for Diagnostic Purposes

The use of CRISPR for molecular diagnosis has provided another exciting development during the last couple of years. Initially, the promiscuous ribonuclease activity of Cas13a upon target recognition of the surrounding RNA molecules was used to develop a molecular detection platform termed specific high-sensitivity enzymatic reporter unlocking (SHERLOCK). This platform was used for the detection of the Zika and Dengue viruses [125]. Moreover, a new version of SHERLOCK (SHERLOCKv2) was developed that can simultaneously detect multiple targets in the same reaction using different Cas enzymes and fluorescent reporters [126]. The SHERLOCK system was also shown to identify single-nucleotide polymorphisms (SNPs) on the viral genome [127]. Similarly, Cas12a possesses a non-specific cleavage of ssDNA molecules upon recognition of targeted sequence; using this feature, a DNA detection platform termed DNA Endonuclease Targeted CRISPR Trans Reporter (DETECTR) was developed [128]. Interestingly, both SHERLOCK and DETECTR approaches have been used for the detection of SARS-CoV-2 from nasal swabs during the recent COVID19 pandemic [129]. Notably, in the case of both the approaches, the results can be obtained in less than one hour and can be observed on a lateral flow strip. Furthermore, SHERLOCK and DETECTR methods are specific and sensitive in detecting the nucleic acids at low concentrations. These features make them ideal as care diagnostic tools that can be deployed and used with minimal setup during pandemics such as COVID19. 

Interestingly, the RNA targeting property of CRISPR was also tested to target the SARS-CoV-2 genome. A CRISPR-Cas13-based strategy, PAC-MAN (prophylactic antiviral CRISPR in human cells), was shown to target and degrade RNA from SARS-CoV-2 sequences and H1N1 IAV load in respiratory epithelial cells [130]. In this study, the PAC-MAN was tested in an in vitro setting; however, the successful translation of this approach in vivo requires a non-viral delivery vehicle. Nanoparticles can be used to deliver mRNAs encoding CasRx/gRNAs directly into the lungs in an aerosol format using a nebulizer, are an ideal choice. Nevertheless, directly targeting the virus RNA genome is a promising strategy to prevent viral replication without relying on the body’s immune system, which is an important factor for patients with low immunity or older individuals that cannot fight the infection and develop antibodies efficiently [131,132]. 

## 6. Gene Editing in Human Embryos

Genetic mutations in the germline of parents are passed down to the next generation. Some of the mutations can be lethal and affect embryo development that may lead to the early termination of pregnancy. In some less severe cases, the mutation can lead to the development of disease later during life. With the advancements in diagnosis and availability of cutting-edge medical interventions, it is now possible to treat or prevent the progression of many diseases. However, we still lack effective methods for treating the majority of the diseases caused by the genetic mutations inherited from the parents, so the correction of these mutations in the early stage of embryo development has been considered. Nevertheless, genome editing in somatic cells and germline differs greatly, not only in terms of DNA repair mechanism, but also in long-term consequences. Genome editing in somatic cells involves the modification of patient cells to cure a disease, which can be performed by isolating the cells and transplanting them back after correcting the mutation. However, for germline editing, the correction needs to be performed during the early stages of embryo development, and all the cells from the embryo may carry the modification, including the germ cells, which will also impact the future generations. Alternatively, it is possible that only some cells in the embryo undergo correction, which can lead to the generation of a mosaic embryo.

In the last few years, several research groups have performed gene editing in human embryos. Until now, more than seven different studies have been carried out using human embryos to test the cleavage efficiency and off-targets using CRISPR-Cas system [133,134,135,136]. Interestingly, during the correction of a pathogenic mutation in human embryos, DSBs have been repaired using endogenous HDR mechanism and wild-type allele as a template, which differs from the HDR efficiency observed in pluripotent stem cells [135]. In addition to the correction of pathogenic mutations, gene editing has also been used performed in human embryos to understand the role of pluripotent transcription factor OCT4 during early embryogenesis [137]. Moreover, base editing technology has also been used in the human embryos to correct pathogenic mutations [138,139]. Interestingly, better correction efficiency and higher homozygotic nucleotide conversion with no overlapped mutations have been observed at two-cell and four-cell human embryos compared to zygote [139].

Interestingly, the development of a long-term in vitro culture system for human embryos (until 14 days) [140,141], opened up possibilities of culturing the gene-edited embryo to better understand the early developmental problems. Moreover, pluripotent stem cells are now cultured in vitro on different cellular matrices to allow them to self-organize and generate structures called synthetic embryos that are similar to normal embryos and mimic the early developmental program of natural embryos [142]. Recently, using a single stem cell type, extended pluripotent stem cells of mouse origin, and a 3D-differentiation system has been used to generate blastocyst-like structures [143]. A similar approach is currently being refined to create synthetic embryos using human pluripotent stem cells. We can foresee that in the near future, the synthetic embryos might help to replace the use of natural human embryos for basic research purposes, especially for gene editing, to generate various disease models. Notably, the successful generation of a synthetic human embryo can, to some extent, avoid the ethical issues surrounding the use of human embryos for basic research purposes [144,145].

Until recently, the goal of genome editing in human embryos was intended to better understand the efficacy of gene correction and early developmental problems without implanting the edited embryos. However, to prevent HIV infection, one researcher in China to prevent HIV infection attempted to modify the *CCR5* gene in the human embryos that were later transferred to a human resulting in the birth of twin babies. This controversial experiment reignited an international debate on the necessity and ethical issues on genome editing in human embryos. Currently, in several different countries, a moratorium exists on genome editing in human embryos for clinical purposes. According to the guidelines developed by the National Academy of Science, clinical trials for heritable genome editing can be permitted when performed adhering to the regulatory framework and fulfilling a list of criteria that includes, among others things, the absence of reasonable alternatives, the prevention of the transmission of serious disease, the restriction of the conversion of genes to the versions already prevalent in the population, plans for long-term and multigenerational follow-up, and oversight to prevent the use for other purposes [146,147]. Reports from the World Health Organization and International Commission on the Clinical Use of Human Germline Genome editing organized by US National Academy are due later this year. In general, pre-implantation genetic diagnosis (PGD) can be used to select un-mutated embryos free of mutation for implantation and avoid genome editing. However, the selection-based approaches can be a challenge for families who produce a lower number of embryos or when one of the partners carries a homozygous autosomal dominant mutation [148].

## 7. Future Perspectives

Customizable nucleases have opened new possibilities in the treatment of genetic mutations. Promising results from in vitro experiments and animal models demonstrate the potential application of CRISPR-Cas system in both basic research and clinical settings. However, before these nucleases can be used in clinics, several improvements must be achieved, including more precise targeting efficiency, lower off-targets, fewer unintended changes and a good delivery vehicle that can target a wide range of tissues when delivered in vivo. Certainly, significant progress on these requirements has been made. Notably, the repair efficiency has been improved by delivering Cas protein, and the availability of different types of Cas nucleases isolated from other prokaryotes and nickases are used to avoid DSBs and reduce the off-target activity [31]. Moreover, to prevent the unintended changes at the targeting site during the correction, the intronic region upstream of the mutation site is targeted to perform gene knock-in; this approach will offer greater flexibility in designing the gRNAs, and any additional changes will potentially not affect the gene function [38]. Similarly, the use of delivery vehicles other than viruses, including nanoparticles, are being tested to deliver the CRISPR-Cas system efficiently and safely. Another factor that might affect the clinical use of CRISPR-Cas system is the pre-existing antibodies against Cas proteins due to their bacterial origin, which could lead to inflammation and lower stability of Cas proteins [31]. However, more evidence is needed to determine the minimal levels of Cas protein that can activate the immune system. Together, these improvements will aid in using the CRISPR-Cas system to correct the mutations in vivo and cure the genetic disease. Notably, people with some genetic variants are more susceptible to the development of neurological disorders and some cancers. Moreover, with age, the number of mutations in somatic cells increases dramatically, and some of these mutations can lead to the development of cancer [149]. The availability of an efficient CRISPR-Cas system might facilitate the modification of specific regions of the genome in the future to prevent the development of diseases, for example, a variant of the gene that encodes for less functional protein or familial mutation that can lead to the development of the neurodegenerative disease. Moreover, the development of efficient genome editing tools, including the base editors with high specificity and no off-target effects, will open the possibilities of using them in human embryos to avoid the transmission of the disease-causing mutation. Similarly, the newly developed CRISPR-free mitochondrial base editing approach promises tremendous potential in the future to correct the pathogenic mutations in the mtDNA present in the unfertilized oocytes or embryos and prevent the transmission of mitochondrial diseases to the next generation.

In addition to the application of customizable nucleases for gene editing, their use in modulating the expression of the genes by changing the epigenetic marks on the promoters offers great potential. Interestingly, genetic diseases are not only caused due to the mutations in a gene, but also due to the lower expression of said genes that can affect the function of tissues and organs. Notably, during aging, dysregulation of epigenetic marks leads to the decreased expression of several different genes that are important for the normal function of cells and tissue, which eventually leads to the manifestation of disease phenotypes [150,151,152]. Since customizable nucleases were shown to modulate the expression of target gene without modifying the genomic sequences, they can be an attractive method in clinical settings to treat various diseases. Notably, we have shown that the use of CRISPR-Cas system may allow the activation of endogenous genes in vivo and reverse disease phenotypes [108]. In vivo gene activation using a CRISPR-Cas system can also overcome several limitations posed by traditional gene therapy, including the size and number of transgenes that can be delivered. Age-associated disorders are not caused due to dysregulation of a single gene or pathway, and in such cases, multiple genes may need to be activated at the same time. For this reason, a multiplex system needs to be developed in which several gRNAs can be simultaneously delivered to every cell and activate multiple genes that will help to ameliorate the cellular hallmarks of aging and restore the function of the tissues. Furthermore, the use of tissue or cell-specific promoters will help to restrict the expression of Cas enzyme or gRNAs and prevent unintended gene activation in other tissues. 

In conclusion, during the last couple of years, we have not only witnessed the discovery and development of new genome editing approaches, but also their implementation to treat various diseases. Currently, many clinical trials are underway that use the newly developed gene-editing tools, and in the next few years, some of them will be eventually used in clinics not just for the treatments of genetic diseases, but also to prevent or treat viral infections such as SARS-CoV-2.
